# Activation of Sirt1 by Resveratrol Inhibits TNF-α Induced Inflammation in Fibroblasts

**DOI:** 10.1371/journal.pone.0027081

**Published:** 2011-11-01

**Authors:** Xiaoxia Zhu, Qiong Liu, Meimei Wang, Minrui Liang, Xue Yang, Xue Xu, Hejian Zou, Jianhua Qiu

**Affiliations:** 1 Division of Rheumatology, Shanghai Medical College, Fudan University, Huashan Hospital, Shanghai, China; 2 Institute of Rheumatology, Immunology and Allergy, Shanghai Medical College, Fudan University, Huashan Hospital, Shanghai, China; 3 Department of Human Anatomy, Histology and Embryology, Shanghai Medical College, Fudan University, Shanghai, China; 4 Division of Rheumatology, Dongnan University, Zhongda Hospital, Nanjing, China; 5 Department of Neurology and Radiology, Harvard Medical School, Massachusetts General Hospital, Charlestown, Massachusetts, United States of America; Charité-University Medicine Berlin, Germany

## Abstract

Inflammation is one of main mechanisms of autoimmune disorders and a common feature of most diseases. Appropriate suppression of inflammation is a key resolution to treat the diseases. Sirtuin1 (Sirt1) has been shown to play a role in regulation of inflammation. Resveratrol, a potent Sirt1 activator, has anti-inflammation property. However, the detailed mechanism is not fully understood. In this study, we investigated the anti-inflammation role of Sirt1 in NIH/3T3 fibroblast cell line. Upregulation of matrix metalloproteinases 9 (MMP-9), interleukin-1beta (IL-1β), IL-6 and inducible nitric oxide synthase (iNOS) were induced by tumor necrosis factor alpha (TNF-α) in 3T3 cells and resveratrol suppressed overexpression of these pro-inflammatory molecules in a dose-dependent manner. Knockdown of Sirt1 by RNA interference caused 3T3 cells susceptible to TNF-α stimulation and diminished anti-inflammatory effect of resveratrol. We also explored potential anti-inflammatory mechanisms of resveratrol. Resveratrol reduced NF-κB subunit RelA/p65 acetylation, which is notably Sirt1 dependent. Resveratrol also attenuated phosphorylation of mammalian target of rapamycin (mTOR) and S6 ribosomal protein (S6RP) while ameliorating inflammation. Our data demonstrate that resveratrol inhibits TNF-α-induced inflammation via Sirt1. It suggests that Sirt1 is an efficient target for regulation of inflammation. This study provides insight on treatment of inflammation-related diseases.

## Introduction

Autoimmune diseases such as rheumatoid arthritis and systemic sclerosis are characterized by aseptic inflammation manifested with upregulation of pro-inflammatory cytokines [1], [2]. Increase of cytokines further enhances and sustains inflammatory processes and causes tissue damage [3], [4]. Inhibition or neutralization of cytokines suppresses inflammatory cascades and improves functional recovery in experimental models [5], [6]. In clinic, blockade of TNF-α by anti-TNF-α antibodies notably reduces inflammation and ameliorates clinical outcomes [7], [8], [9], [10], suggesting that cytokines play a central role in autoimmune diseases. Reduction of cytokines production or suppression of their signaling is an efficient therapeutic target.

Sirtuin 1 (Sirt1), a mammalian homolog of Sir2, is a NAD^+^-dependent class III histone deacetylase. It has been shown to be involved in a variety of pathophysiological processes, such as anti-inflammation, cell growth and metabolism modulation, anti-carcinogen [11], [12], [13]. Sirt1 regulates pro-inflammatory mediator [14], [15], [16]. Knockout or knockdown of Sirt1 gene leads to increase of cytokines release whereas Sirt1 activation by its activators inhibits productions of TNF-α, monocyte chemoattractant protein 1 (MCP-1) and IL-8 [14], [15], [16]. Furthermore, Sirt1 has inhibitory effects in experimental chronic inflammatory diseases such as chronic obstructive pulmonary disease and colitis [14], [16], [17]. Suppression of pro-inflammatory cytokines production by Sirt1 is highly related to its negative regulation of NF-κB activity by deacetylating of RelA/p65 subunit at lysine 310 [15].

Resveratrol (trans-3,4′,5-trihydroxystilbene), a polyphenolic phytoalexins, is a potent activator of Sirt1 [18]. Increase of evidence indicates that resveratrol exerts an anti-inflammatory property [19], [20], [21]. Resveratrol has a chondroprotective capacity through suppressing the production of IL-1β and reactive oxygen species (ROS) [22]. In human primary airway epithelial cells, resveratrol inhibits cytokine-stimulated iNOS expression and nitrite production [23]. Resveratrol also protects cartilage against the development of experimentally induced inflammatory arthritis [24]. Recent multiple lines of evidence demonstrate that resveratrol inhibits inflammation via blockade of NF-κB transcriptive activity [25], [26], [27]. Resveratrol decreases the expression of NF-κB subunit RelA/p65 or attenuates translocation of p65 from the cytosol to the nucleus with stabilization of inhibitory IκB, and further downregulates levels of TNF-α and cyclooxygenase-2 (COX-2) [19], [28].

Sirt1 may be a promising target for anti-inflammation therapy [29]. In the present study, we investigated the inhibitory role of Sirt1 in TNF-α induced cytokine production in fibroblast cells through activating Sirt1 with resveratrol or downregulating Sirt1 by RNA interference. We further demonstrated that resveratrol inhibited inflammation via a Sirt1-dependent manner.

## Methods

### Cell culture and treatment

Mouse embryonic 3T3/NIH fibroblasts (obtained from the American Type Culture Collection) were cultured in Dulbecco's Modified Eagle's Medium (DMEM) supplemented with antibiotics (100 U/ml penicillin, 100 µg/ml streptomycin) and 10% fetal bovine serum, at 37°C in a humidified incubator with 5% CO_2_. Recombinant mouse TNF-α (R&D System), resveratrol (Sigma) or rapamycin (EMD4Bioscience) were used in this study.

### Gelatin zymography

Gelatin zymography was done as previous described [30], [31]. Briefly, culture media were collected after treatment and subjected to SDS-PAGE in 10% polyacrylamide gels copolymerized with 1 mg/ml gelatin. After electrophoresis, gels were washed in renature buffer to remove the SDS and further incubated with developing buffer (Invitrogen) at 37°C for 24 hours. The gels then were stained with Coomassie blue R-250 (Bio-Rad) for 15 minutes followed by destaining in deionized water with 10% acetic acid and 20% methanol. MMP-9 expression and proteolytic activity were evidenced as clear bands against the background of stained gelatin.

### Western blotting

Cells were lysed in RIPA buffer (50 mM Tris-Hcl pH 7.4, 150 mM NaCl, 1% NP-40, 0.5% sodium deoxycholate, 0.1%SDS, proteinase inhibitor (Roche) and phosphatase inhibitor (Calbiochem) ). Protein concentration was detected using the DC™protein assay (Bio-Rad). Protein (30 µg) was loaded to 10% SDS-PAGE and semi-dry transferred onto a Polyvinylidene fluoride (PVDF) membrane. After blocking with 5% non-fat milk, membranes were incubated overnight at 4°C with primary antibodies against IL-1β (1∶500, Santa Cruz), Sirt1(1∶500, Millipore), acetyl-NF-κBp65(Lys310) (1∶500, Cell Signaling), phosphor(ser245/236)-S6RP (1∶1000, Cell Signaling), phosphor(ser2448)-mTOR (1∶1000, Cell Signaling). Horseradish peroxidase-conjugated secondary antibodies were used for ECL-plus (GE Healthcare) detection. The results were normalized to β-actin (1∶5000, Abcam).

### Real-time RT-PCR

Total RNA was extracted using RNAspin Mini Isolation Kit (GE Healthcare), and reverse-transcribed into cDNA using the SuperScripts III First-Strand Synthesis System (Invitrogen). The genes were examined by real-time PCR. Primers were purchased from Applied Biosystems (MMP-9, Mm00442991_m1; IL-6, Mm00446190_m1; iNOS, Mm00440502_m1). Endogenous 18S ribosomal RNA was used as internal control for normalizing gene expression. Results then were assessed by t-test or ANOVA; *P*≤0.05 was considered to be statistically significant.

### RNA interference

siRNA for mouse Sirt1 (ON-TARGETplus SMARTpool) was purchased from Thermo Fisher Scientific and diluted in RNase-free double distilled water (DDW). Cells were seeded on 6-well plates. After 24 hours, cells at 60∼70% confluency were transfected with 20 nM Sirt1 siRNA, 20 nM control siRNA, or the same volume of DDW for 6∼8 hours using Lipofectamine 2000 according to the manufacturer's protocol. Then cells were switched into DMEM and incubated for 72 hours before treatment.

## Results

### TNF-α induced overexpression of MMP-9 and other inflammatory factors in fibroblasts

**Figure 1:**
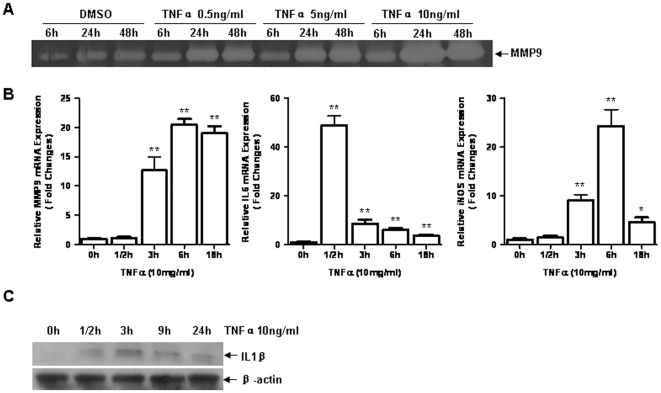
TNF-α induced overexpression of MMP-9, iNOS, IL-6 and IL-1β in 3T3/NIH fibroblasts. A. 3T3/NIH cells were incubated for 6, 24 or 48 hours (h) with varying concentrations of TNF-α, gelatin zymography were performed to detect MMP-9 expression in the medium. **B.** Cells were treated by TNF-α (10 ng/ml) for 0, 1/2, 3, 6, 18 h, relative mRNA expressions of MMP-9, iNOS and IL-6 were examined by real-time RT-PCR analysis (n = 3 per group, *p<0.05 vs. 0 h; **p<0.01 vs. 0 h). **C.** Cells were treated by TNF-α for 0, 1/2, 3, 9, 24 h, IL-1β was measured by western blotting analysis.

TNF-α induces inflammation in various cell types [20], [32]. In current study, we examined expressions of MMP-9, IL-6, iNOS and IL-1β in TNF-α treated 3T3/NIH mouse embryonic fibroblasts. Within 0.5 ng/ml to 10 ng/ml of dose rang, no cell death was observed up to 72 hours after TNF-α treatment (data not shown). As shown in figure 1, TNF-α induced notably MMP-9 upregulation in a dose- and time- dependent manner. The concentration of 10 ng/ml of TNF-α was chosen for investigating the response of fibroblasts to cytokine stimulation. Messenger RNA expressions of MMP-9, iNOS and IL-6 were significantly induced by TNF-α. IL-6 was increased strikingly as early as 30 minutes post treatment; while MMP-9 and iNOS were elevated 3 hours after treatment, and reached to the peak 6 hours after treatment. IL-1β expression was also examined by western blotting analysis. A remarkable upregulation of IL-1β was detected 3 hours after TNF-α treatment. These results are consistent with the reports from other investigators [33], [34].

### Resveratrol blocked TNF-α induced inflammation

**Figure 2:**
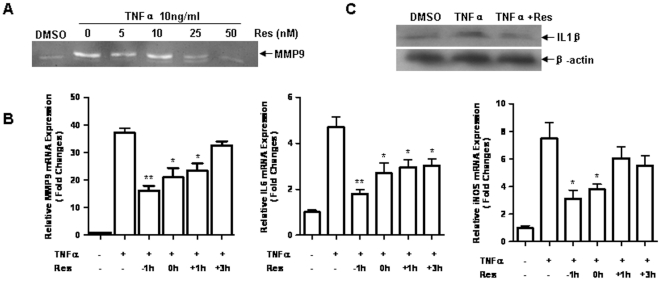
Resveratrol inhibited TNF-α induced inflammation. **A.** 3T3/NIH cells were pretreated 1 h with varying concentrations of resveratrol (Res) before TNF-α (10 ng/ml) treatment. MMP-9 expression in the medium was examined by gelatin zymography after 24 h treatment. **B.** Resveratrol (50 nM) was used to treat cells 1, 0 hour before or 1, 3 hours post TNF-α (10 ng/ml), the relative mRNA levels of MMP-9, iNOS and IL-6 were detected by real-time RT-PCR analysis at 6 hours post TNF-α treatment. (n = 3 per group, *p<0.05 vs. TNF-α+/Res-; **p<0.01 vs. TNF-α+/Res-). **C.** Cells were pretreated by resveratrol 1 h before TNF-α, western blotting analysis was used to detect IL-1β expression after 9 h treatment.

Resveratrol is a potent Sirt1 agonist and increases Sirt1 activity [18]. We employed resveratrol to investigate the anti-inflammation function of Sirt1. 3T3 fibroblasts were pretreated with resveratrol followed by TNF-α challenge. Release of MMP-9 in the culture media was observed in TNF-α treated cells and was greatly inhibited by resveratrol in a dose dependent manner (figure 2A). To test the time course of inflammatory inhibition by resveratrol, resveratrol was administrated 1, 0 hour before or 1, 3 hours post TNF-α treatment, the relative mRNA levels of inflammatory factors were detected by real-time RT-PCR analysis at 9 hours after TNF-α treatment. Resveratrol inhibited the pro-inflammatory factors in a clearly time-dependent fashion (figure 2B). Upregulation of MMP-9, IL-6 and iNOS were attenuated by resveratrol treated when resveratrol was employed before or at the same time of TNF-α treatment. Induced IL-6 upregulation was inhibited even when resveratrol was employed 3 hours after TNF-α stimulation. Interestingly, lack of significant inhibitory effect was observed when resveratrol was treated 1 hour (for iNOS) and 3 hours (for MMP-9) after TNF-α stimulation. Furthermore, the TNF-α induced IL-1β expression was also strikingly inhibited by resveratrol pretreatment for 1 hour (figure 2C). No cell death was observed within 72 hours under these conditions (data not shown).

### Resveratrol Inhibited inflammation via Sirt1

**Figure 3:**
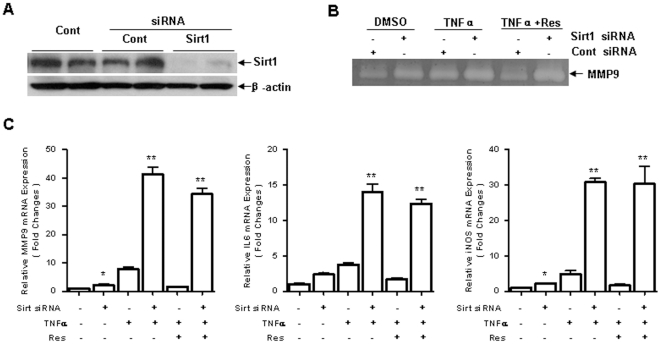
Resveratrol Inhibited inflammation in a Sirt1 dependent manner. **A.** Western blotting analysis shows Sirt1 siRNA (20 nM) transfection for 72 hours strikingly blocked Sirt1 expression, compared to control (Cont) siRNA (20 nM) or DDW transfection. **B.** After Sirt1 or control siRNA transfection for 72 h, cells were h pretreated with resveratrol (50 nM) in the presence of TNF-α (10 ng/ml) treatment for 24 h, MMP-9 expression in the medium were examined by gelatin zymography. **C.** Cells were processed as (B) for 6 h, real-time RT-PCR analysis was used to examine mRNA expression of MMP-9, IL-6, iNOS (n = 3 per group, *p<0.05 vs. Sirt1 siRNA-; **p<0.01 vs. Sirt1 siRNA-).

Since resveratrol is a pharmacological activator of Sirt1 and may have off-target effects, we further examined whether Sirt1 is required for anti-inflammatory activity of resveratrol. Sirt1 expression is induced by TNF-α or inflammation [35]. And Sirt1 activity is highly related to its expression [36], [37]. Here we used RNA interference technique to knock down Sirt1 expression. Expression of Sirt1 was reduced 72 hours after treatment of small interference RNA (siRNA) targeting Sirt1 gene (figure 3A). Inhibitory effect of resveratrol on the upregulation of MMP-9, IL-6 and iNOS was attenuated in the cells in which Sirt1 expression was knocked down (figure 3B-C). These results suggest that anti-inflammation of resveratrol is mainly dependent on Sirt1 and Sirt1 exerts a negative regulatory effect on the inflammation.

### Resveratrol decreased acetylated RelA/p65

**Figure 4:**
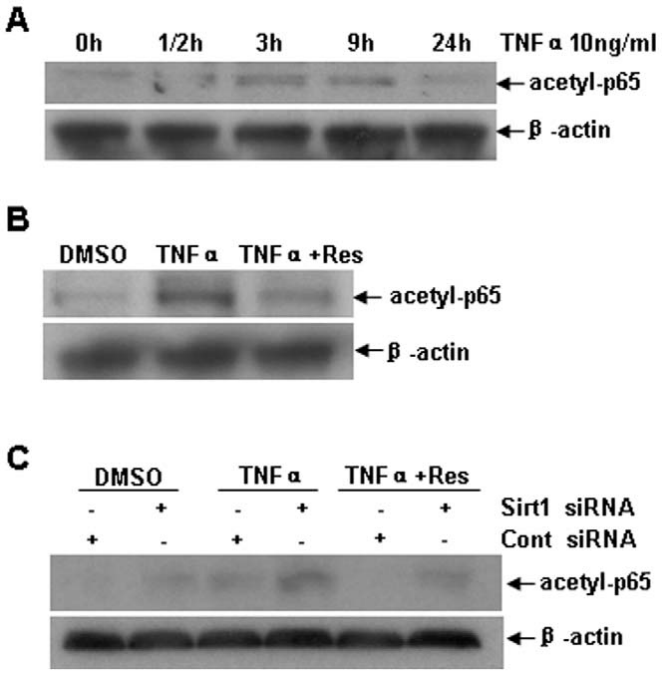
Resveratrol decreased acetylated RelA/p65. A. 3T3/NIH cells were treated with TNF-α (10 ng/ml) for 0, 1/2, 3, 9, 24 h, and acetyl-NF-κBp65(Lys310) (acetyl-p65) expression was detected by western blotting analysis in cell lysis. **B.** Cells were pretreated by resveratrol (50 nM) in the presence of TNF-α treatment for 3 h, acetyl-NF-κBp65(Lys310) expression were examined by western blotting analysis. **C.** Cells were transfected with Sirt1 or control siRNA for 72 h, then pretreated with resveratrol (50 nM) in the presence of TNF-α (10 ng/ml) treatment for 3 h. Western blotting analysis was used to detect acetyl-NF-κBp65(Lys310) expression.

NF-κB plays a central role in inflammation [38]. Acetylasion of NF-κB subunit RelA/p65 at Lysine310 is involved in the activity of NF-κB and inflammatory factors transcription [39]. We found that TNF-α increased acetyl-NF-κBp65 (Lysine310) and resveratrol suppressed NF-κBp65 acetylation in 3T3 cells (figure 4A-B). To explore whether the deacetylatic function of resveratrol is also Sirt1 dependent, Sirt1 was knocked down by siRNA. Interestingly, basal level of acetyl-NF-κBp65 (Lys310) was increased after Sirt1 knockdown. Inhibitory effect of resveratrol on the acetyl-NF-κBp65 was diminished by Sirt1 knockdown (figure 4C). The results indicate that Sirt1 is required for inhibiting NF-κBp65 acetylation by resveratrol. It also suggests that Sirt1 plays a role in regulation of RelA/p65 acetylation.

### Resveratrol inhibited activation of mTOR induced by TNF-α

**Figure 5:**
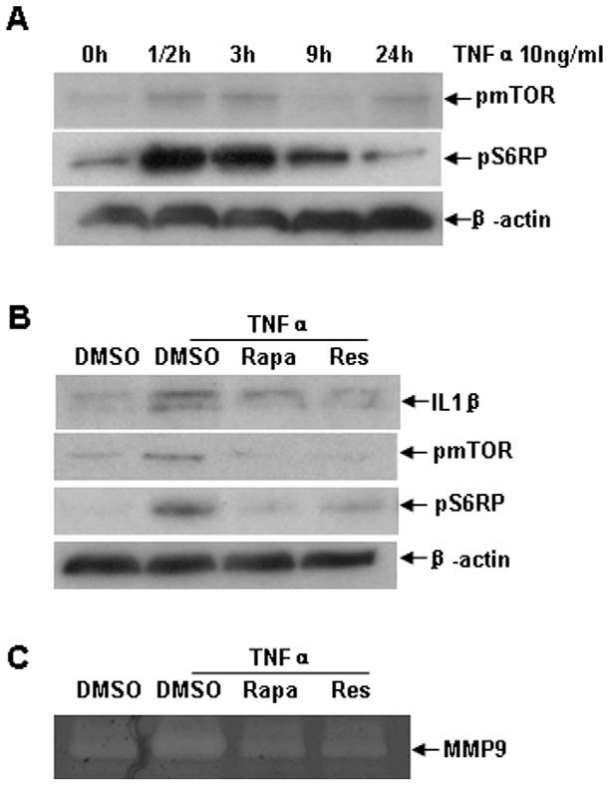
mTOR/S6RP is also involved in the inhibitory function of resveratrol on TNF-α induced inflammation. **A.** 3T3/NIH cells were treated with TNF-α (10 ng/ml) for 0, 1/2, 3, 9, 24 h, phosphorylation of mTOR and S6RP was detected by western blotting analysis. **B.** Cells were pretreated by rapamycin (Rapa, 10 ng/ml) or resveratrol (50 nM) for 1 h in the presence of TNF-α treatment for 24 h, MMP-9 released in medium was measured by gelatin zymography. **C.** Cells were processed as (B) for 3 h, IL-1β and phosphorylation of mTOR and S6RP were examined by western blotting analysis in cell lysis.

Mammalian target of rapamycin is related to TNF-α induced inflammation [40]. In this study, upregulation of phosphorylated mTOR and S6RP were detected as early as 30 minutes and returned back to control level within 24 hours after TNF-α stimulation (figure 5A). We also found that resveratrol suppressed phosphorylated mTOR and S6RP (figure 5B) and further reduced the expression of IL-1β and MMP-9 (figure 5 B–C). Moreover, rapamycin inhibited phosphorylation of mTOR and S6RP, and suppressed MMP-9 and IL-1β upregulation (figure 5 B–C).

## Discussion

Previous studies have revealed that resveratrol activates Sirt1 and suppresses the inflammation of both *in vivo* and *in vitro* experiment models [20], [21], [36], [41]. In the present study, we found that TNF-α induced upregulation of cytokines and MMP-9 in 3T3 fibroblasts. Sirt1 was constitutively expressed in the cells and upregulated after TNF-α treatment. Resveratrol inhibited TNF-α induced overexpression of cytokines, iNOS and MMP-9 via Sirt1 dependent manner. Knockdown of Sirt1 caused increase of IL-6, iNOS and MMP-9 expression. The anti-inflammation function of resveratrol was blocked after Sirt1 knockdown. This study suggests that resveratrol ameliorates inflammation via activating Sirt1. Reduction of acetylated NF-κB and suppression of mTOR/S6RP phosphorylation may be involved in the mechanisms.

TNF-α has been demonstrated to be one of the main inflammatory mediators that involved in autoimmune diseases, such as rheumatoid arthritis, systemic sclerosis [2], [42]. TNF-α initiates or aggravates inflammation by activating NF-κB and producing cytokines, chemokines, MMPs, and other inflammatory molecules in different types of cells [20], [32]. In the present study, we found that TNF-α induced MMP-9, IL-6, and iNOS in a dose- and time-dependent fashion without causing cell death in fibroblasts. These findings are consistent with other reports [20], [21]. We applied this *in vitro* model to further investigate the role of Sirt1 in TNF-α induced inflammation.

Sirt1 plays a critical role in regulation of several transcription factors such as p53, NF-κB and FoxOs and has many important functions in metabolism, anti-cancer, anti-ageing and anti-inflammation [12], [13], [43], [44]. Overexpression of Sirt1 or increase of Sirt1 activity significantly suppresses cytokines production and reduces inflammation in different animal models [20], [21]. On the other hand, reduction of Sirt1 activity results in increase of inflammatory response [45], [46]. It has been shown that TNF-α induces inflammation through activation of cathepsin B followed by cleavage of Sirt1 [47]. More severe encephalomyelitis and autoimmunity induced by myelin oligodendroglia glycoprotein (MOG) peptide is detected in Sirt1-deficient mice compared to the controls [48]. MMP-9 expression in Sirt1-/- mouse embryonic fibroblasts (MEF) is significant higher than that in wild type cells [49]. Our data showed that Sirt1 was constitutively expressed in the 3T3 fibroblasts (figure 3A). Knockdown of Sirt1 caused increase of MMP-9, iNOS and IL-6 expression. Similar phenomenon is also observed in vascular smooth muscle cells. The inhibitory effect on AP-1, c-Jun or c-Fos by Sirt1 might be the potential mechanism [48], [50]. These results suggest that Sirt1 plays a role in suppressing expression of inflammatory molecules even under normal conditions.

Resveratrol is a potent activator of Sirt1 and has multiple effects on metabolism, anti-cancer, anti-ageing and anti-inflammation [11], [19], [51], [52]. Treatment of resveratrol suppresses cyclooxygenase-2 activity [53] and modulates interferon-gamma (IFN-γ), TNF-α, IL-6 and MCP-1 expression in experimental ileitis [54]. In cultured neural cells, resveratrol prevents increase of cytokines, chemokines and iNOS/NO induced by lipopolysaccharide (LPS) [55]. In IL-1β treated human chondrocytes, resveratrol inhibits the cytokine production [22]. Furthermore, resveratrol ameliorates LPS induced inflammatory arthritis *in vivo* [24]. In this study, we observed that resveratrol significantly inhibited the TNF-α induced increase of MMP-9, IL-6, iNOS and IL-1β in the fibroblasts. The inhibitory effect was in a dose- and time-dependent fashion. The evidence indicates that resveratrol is an efficient inhibitor of inflammation in fibroblasts.

Although resveratrol is a pharmacological agonist of Sirt1 and may have multiple targets, several studies indicate that the anti-inflammatory function of resveratrol is highly dependent on Sirt1 [51], [56]. Resveratrol upregulates Sirt1 expression at both transcriptional and translational level [57], [58]. Furthermore, by binding to Sirt1, resveratrol alters Sirt1 structure and upregulates its activity as much as 8 folds, and lowers the *K*_m_ value for acetylated substrate [11], [18]. However, other studies suggest that resveratrol may not directly activate Sirt1 [59], [60]. Resveratrol lacks inhibition of upregulation of hypoxia induced factor 1 alpha (HIF-1α) and AMP-activated protein kinase (AMPK) in the cells in which Sirt1 is deficit [61]. Reduction of MMP-9 expression by resveratrol is remarkably attenuated in Sirt1-/- mouse embryonic fibroblasts (MEF) [49]. But the inhibitory effect of resveratrol on Smad2/3 phosphorylation was reported to be independent of Sirt1 [62], [63]. In the present study, suppression of TNF-α-induced inflammatory response by resveratrol was attenuated in the cells in which Sirt1 was knocked down by RNA interference. Our findings support that Sirt1 is required for anti-inflammation of resveratrol and suggest that Sirt1 is an important target for treatment of autoimmune disorders.

Several lines of evidence indicate that NF-κB is one of Sirt1 targets. NF-κB acetylation is critical for its activity, especially in cytokines induced inflammation [64]. Acetylation of NF-κB at specific lysine residues results in uncoiling of the DNA and increased accessibility to transcription factor binding which contributes to pro-inflammatory factors production [65], [66]. Sirt1 physically interacts with the RelA/p65 subunit of NF-κB and inhibits transcription by deacetylation of RelA/p65 at lysine 310 [15], [67]. Acetylation of RelA/p65 at lysine 310 has been reported to be required for full transcriptional activity of NF-κB in inflammation [68]. In this study, we found that TNF-α increased acetylation at Lysine 310 of RelA/p65 subunit. Resveratrol inhibited the TNF-α induced acetylation of RelA/p65 via a Sirt1-dependent manner (figure 4). Our findings further support the elucidation of the mechanism that the inhibitory effect of Sirt1 is due to suppressing NF-κB activity.

Mammalian target of rapamycin (mTOR) is a serine/threonine protein kinase which regulates protein synthesis [69], [70]. Studies report that activation of mTOR plays a role in TNF-α-induced inflammatory cascades [71], and is also implicated in inflammation related diseases [72]. TNF-α increases phosphorylation of mTOR and its downstream targets p70S6K and S6RP [73], [74], [75]. Rapamycin ameliorates inflammation by inhibiting mTOR and further decreasing expression of cytokines and chemokines, improves outcomes in inflammation related disease models [76], [77]. These results suggest that mTOR is an important target for treatment of inflammation. In the present study, mTOR and S6RP were phosphorylated and activated in TNF-α stimulated fibroblasts. Resveratrol also notably downregulated the phosphorylated mTOR and S6RP while ameliorating the inflammation in fibroblasts, suggesting that inhibition of mTOR may be one of the mechanisms of its anti-inflammatory effect.

In summary, our results indicate that Sirt1 plays a key role in inflammation. Activation of Sirt1 by its agonists such as resveratrol efficiently suppresses inflammatory cascades. This study provides an insight for developing therapeutic approaches for inflammation related diseases or autoimmune disorders. Further investigation in animal models is warranted to confirm the findings from *in vitro* studies.
